# Maturational and Aging Effects on Human Brain Apparent Transverse Relaxation

**DOI:** 10.1371/journal.pone.0031907

**Published:** 2012-02-21

**Authors:** Jianli Wang, Michele L. Shaffer, Paul J. Eslinger, Xiaoyu Sun, Christopher W. Weitekamp, Megha M. Patel, Deborah Dossick, David J. Gill, James R. Connor, Qing X. Yang

**Affiliations:** 1 Department of Radiology, Pennsylvania State University College of Medicine, Hershey, Pennsylvania, United States of America; 2 Department of Public Health Sciences, Pennsylvania State University College of Medicine, Hershey, Pennsylvania, United States of America; 3 Department of Neurology, Pennsylvania State University College of Medicine, Hershey, Pennsylvania, United States of America; 4 Department of Neurosurgery, Pennsylvania State University College of Medical, Hershey, Pennsylvania, United States of America; Banner Alzheimer's Institute, United States of America

## Abstract

The goal of this study was to address the need for comprehensive reference data regarding maturational and aging effects on regional transverse relaxation rates (R_2_) of the brain in normal humans. Regional R_2_s were measured in twenty-five brain structures from a sample of seventy-seven normal volunteers 9 to 85 years of age. The relationships between regional R_2_ and age were determined using generalized additive models, without the constraint of a specified *a priori* model. Data analysis demonstrated that the brain tissue R_2_-age correlations followed various time courses with both linear and non-linear characteristics depending on the particular brain structure. Most anatomical structures studied exhibited non-linear characteristics, including the amygdala, hippocampus, thalamus, globus pallidus, putamen, caudate nucleus, red nucleus, substantia nigra, orbitofrontal white matter and temporal white matter. Linear trends were detected in occipital white matter and in the genu of corpus callosum. These results indicate the complexity of age-related R_2_ changes in the brain while providing normative reference data that can be utilized in clinical examinations and studies utilizing quantitative transverse relaxation.

## Introduction

The transverse relaxation time (T_2_) and transverse relaxation rate (R_2_), where R_2_ = 1/T_2_, play a fundamental role in generation of MRI contrast in the human brain. Quantitative T_2_ and R_2_ brain mapping has been used in studies of various neurological disorders across a wide age range, e.g., white matter abnormalities, brain tumors, schizophrenia, multiple sclerosis, autism, and Alzheimer's disease [1], [2], [3], [4]. Previous studies on brain transverse relaxation have shown a general trend of T_2_ decrease during maturation while, conversely, showing a T_2_ increase during aging [5], [6], [7], [8], [9], [10], [11], [12], [13], [14], [15], [16], [17], [18]. Despite these trends, the T_2_-age correlation in the human brain is not yet well characterized, making interpretation of deviations from normative values uncertain.

Saito et al. studied 18 normal volunteers and 33 patients with either no or small brain lesions at 1.5 T and showed that T_2_ for the brain falls into four distinct periods of life: 0–2 years old (maturation period), 2–20 years old (development period), 20–60 years old (adulthood period) and ≥60 years (senescence period) [8]. Most of the subjects in this study, however, had brain disorders, raising the concern that the reported T_2_ values do not represent truly normal findings. A study at 1.5 T by Siemonsen et al. on 50 patients (12–91 years of age) with no significant brain lesions except white matter leukoaraiosis, found an increase in T_2_ that linearly correlated with age in the thalamus and three white matter structures, but not in the caudate nucleus and lentiform nucleus [9]. Another study on 70 normal subjects aged 3 weeks to 31 years showed that T_2_ decreased with increasing age; the rate of decrease was greater at a younger age and slower in the years after [10], indicating a nonlinear relationship with age. Kim et al. studied the corpus callosum in 33 normal pediatric subjects aged 3–15 years at 3 T and reported a significant T_2_- age correlation in the splenium, but not in the genu [11]. After studying 33 normal subjects aged 19–59 years at 3 T, Hasan et al. did not find significant T_2_-age correlation in the caudate nucleus [12]. The discordant findings of these previous studies demonstrate the need for establishing a more comprehensive T_2_ mapping data set, based on a larger normal cohort with a greater age range and more brain structures.

The analytic approaches used in the previous studies of T_2_-age correlations have been based on *a priori* models. Most of them have employed linear regression [9], [11], [12], [13], [18]. Hasan et al. reported using both linear and quadratic terms to estimate the aging effects on T_2_ and the relation between T_2_ and age in whole brain gray and white matter, caudate nucleus, and the anterior limb of internal capsule followed a quadratic, U-shaped curve [17]. Although plausible, little histopathological data is available to support these *a priori* models. Therefore, the current study was designed to address these limitations and elucidate the effects of development and aging on regional T_2_/R_2_ in the normal human brain without the bias of *a priori* models. In this study, we employed generalized additive models (GAM), a well-known nonparametric approach to regression that can accommodate any potential nonlinear relationship [19]. The goals of this study were: 1) to establish standardized, normative T_2_ maps of several age intervals as references for clinical trials and routine examinations, 2) to determine continuous developmental and aging characteristics in representative brain structures, and 3) to determine the regional T_2_ differences among these brain structures.

## Methods

Seventy-seven volunteers without known neurologic or psychiatric disorders aged 9 to 85 years (41 males and 36 females) participated in the study (see Table 1). There was no significant difference between the age distributions in the two genders. To exclude abnormal cognitive disorders, the 39 subjects over the age of 50 (average education 14.9±1.6 years) received the Mini-Mental State Examination (MMSE) [20] and the Clinical Dementia Rating Scale (CDR) [21]. All 39 subjects had a CDR score of 0, meaning fully oriented; their average score on the MMSE was 29.1±1.0, which is in the normal range of 25 to 30. The study protocol was approved by the Penn State Hershey Institutional Review Board. All subjects or parents of subjects under 18 years old gave informed, written consent prior to participation.

**Table 1:** Age distribution of the study cohort.

Age (year)	Number of Subjects
9–12	6
13–19	8
20–29	14
30–39	6
40–49	4
50–59	16
60–69	9
70–79	6
>80	8

The T_2_/R_2_ mapping was acquired on a 3 T scanner (Bruker MedSpec S300 with TEM head coil, Bruker BioSpin Corporation, Ettlingen, Germany) with maximal strength 3 gauss/cm using a 9-echo spin-echo sequence with TE from 11.8 to 106.2 ms (TR = 4000 ms, flip angle = 180°, Gaussian radio-frequency (RF) pulse, bandwidth = 80 kHz, 20 2.5-mm-thick axial slices with no gap between slices centered at hippocampus, FOV = 25×25 cm^2^, acquisition matrix = 256×192, reconstruction matrix = 256×256, number of average = 1). A test-retest was performed on five normal young subjects (all male, 29.0±3.3 years of age), who received two back-to-back brain T_2_/R_2_ mapping protocol on the same magnet. No significant difference was observed between R_2_s obtained from the two scans (paired t-test, p>0.20).

**Figure 1:**
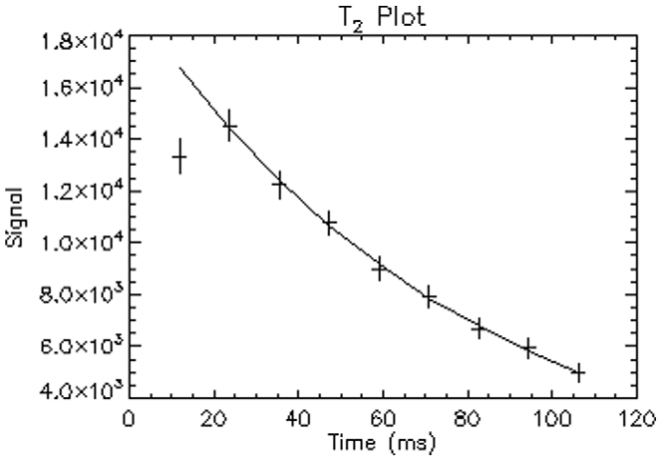
Example T_2_ relaxation regression plot of left putamen from a healthy 33-year-old man. The plus signs are the signal intensity; the solid line is the fitted curve for the T_2_/R_2_ estimation (R^2^ = 0.999).

The R_2_ maps were generated using linear regression of the logarithm of the signal intensity:
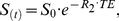
(1)with custom-designed software qMRI (http://pennstatehershey.org/web/nmrlab/resources/software/qmri) written with Interactive Data Language (Research Systems, Inc., Boulder, CO). Figure 1 shows an example of the T_2_ relaxation regression plot. As indicated in the figure, the first echo of the echo train had a significant contribution from a stimulated-echo that depended on T_1_ of the tissue for a given TR and, thus, was excluded from the T_2_/R_2_ estimation. The resultant spatial resolution of the R_2_ map was 1×1×2.5 mm^3^. Then the R_2_ maps were normalized to the Montreal Neurological Institute brain template [22] using SPM5 (Wellcome Trust Centre for Neuroimaging, University College London, UK) [23]. The normalized R_2_ maps had a spatial resolution of 1×1×2.5 mm^3^. These data are available on-line at http://www.pennstatehershey.org/web/nmrlab/resources1.

**Figure 2:**
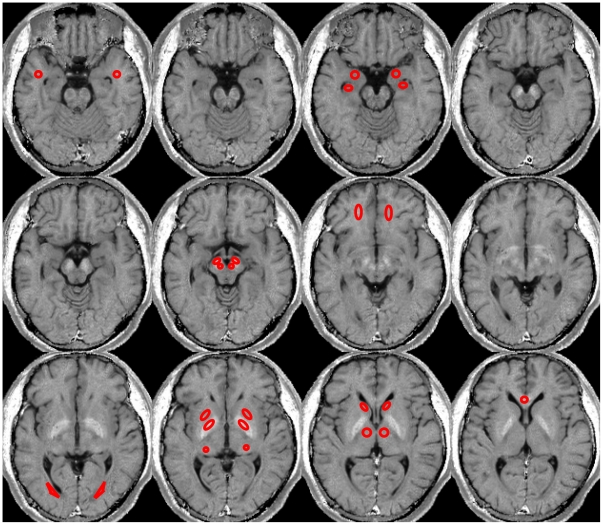
ROIs shown on a normalized R_2_ map from a healthy 33-year-old man. ROIs include: amygdala, head of hippocampus, anterior thalamic nucleus, posterior thalamic nucleus, genu of globus pallidus, putamen, head of caudate nucleus, red nucleus, substantia nigra, genu of corpus callosum, orbitofrontal white matter, anterior temporal white matter, and occipital white matter.

Regional R_2_s were obtained from manually drawn regions of interest (ROI) in twenty-five brain structures as shown in Figure 2. These structures were chosen because they are: 1) functionally important and well-studied; 2) clinically important as they involve in many neurological disorders such as Alzheimer's disease and Parkinson's disease; and 3) they are relatively homogeneous structurally and have clear boundaries with neighboring structures. Cortical gray matter was not studied because of significant partial volume effects from subcortical white matter and cerebrospinal fluid signal on the surface of the brain. In order to select regions of interest (ROIs) that were representative of given brain structures with minimal contaminations of surrounding tissues, the following rules were followed: 1) an ROI should be in the center of the structure where the R_2_ distribution is relatively homogeneous; 2) tissues surrounding an ROI in the two adjacent slices should be within the same structure; and 3) an ROI should be at least one voxel away from surrounding structures in the image plane. Eighteen gray matter and seven white matter structures with clear boundaries from surrounding structures were selected from the R_2_ maps, as illustrated in Figure 2. They are the amygdala, head of hippocampus, genu of corpus callosum, anterior and posterior nucleus of thalamus, head of caudate nucleus, globus pallidus, putamen, substantia nigra, red nucleus, orbitofrontal white matter, anterior temporal white matter, and occipital white matter. The size of ROIs varied from 21 voxels (e.g., red nucleus) to 107 voxels (e.g., the anterior nucleus of thalamus) in order to provide representative values for the given brain structures.

The relationship between R_2_ and age was examined with generalized additive models [19]. These models allow the mean of the dependent variable (R_2_) to depend on an additive predictor (age) through a nonlinear link function and are especially useful for visualizing the relationship between a dependent variable and one or more independent variables. The specific GAM employed extends simple linear regression by expanding the linear form of the expected value of the dependent variable:
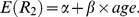
(2)Thus, the model relating R_2_ to age can be expressed as:
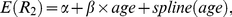
(3)where α is the intercept, β is the slope, and *spline*(*age*) is the partial smoothing spline term.

Plots of partial predictions *spline*(*age*), the estimated smoothing spline function, versus age along with a 95% confidence band were used to assess where nonlinearities occurred between R_2_ and age. If the 95% confidence limits cover the zero axis of the independent variable, it indicates that the nonlinear component of age is not significant. The shape of the plot of partial spline predictions indicates the form of the functional relationship between R_2_ and age. For example, a quadratic shape of the plot would indicate a quadratic relationship between R_2_ and age. Additionally, plots of the prediction of R_2_ overlaid with the observed data allow assessment of the goodness of fit. All models were fit using the GAM procedure in SAS (SAS Institute, Inc. Cary, NC, USA).

## Results

**Figure 3:**
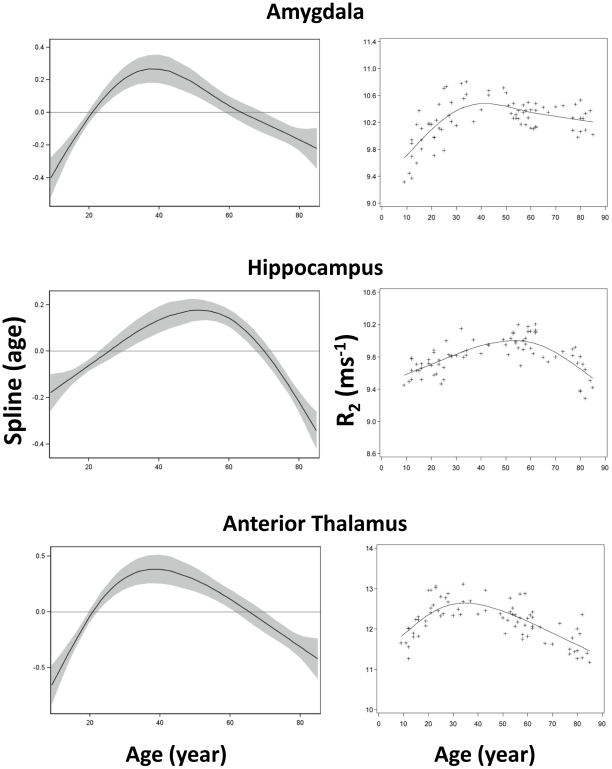
Scatter plots and fitted curves of R_2_-age correlations in amygdala, hippocampus and anterior thalamus. Graphs in the left column are partial predictions plots of estimated smoothing spline functions against age with a 95% confidence band for the whole curve; graphs in the right column plot the predicted values of R_2_ against age (solid line) with the observed data overlaid (plus signs). Top, amygdala; middle, head of hippocampus; bottom, anterior thalamic nucleus.

**Figure 4:**
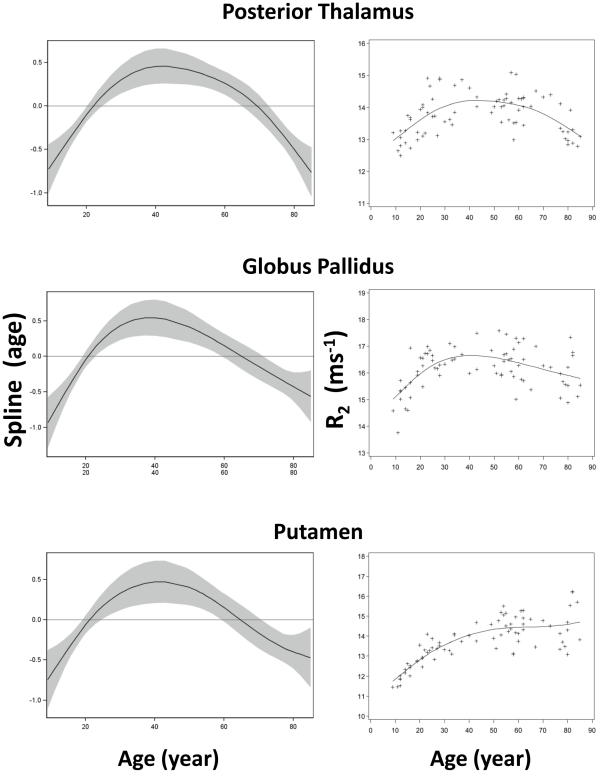
Scatter plots and fitted curves of R_2_-age correlations in posterior thalamus, globus pallidus and putamen. Graphs in the left column are partial predictions plots of estimated smoothing spline functions against age with a 95% confidence band for the whole curve; graphs in the right column plot the predicted values of R_2_ against age (solid line) with the observed data overlaid (plus signs). Top, posterior thalamic nucleus; middle, globus pallidus; bottom, putamen.

**Figure 5:**
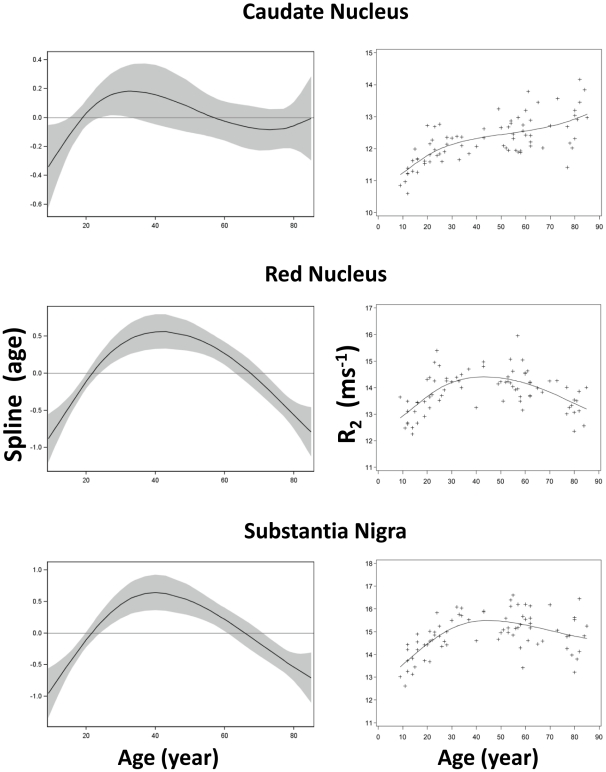
Scatter plots and fitted curves of R_2_-age correlations in caudate, red nucleus and substantia nigra. Graphs in the left column are partial predictions plots of estimated smoothing spline functions against age with a 95% confidence band for the whole curve; graphs in the right column plot the predicted values of R_2_ against age (solid line) with the observed data overlaid (plus signs). Top, head of caudate nucleus; middle, red nucleus; bottom, substantia nigra.

**Figure 6:**
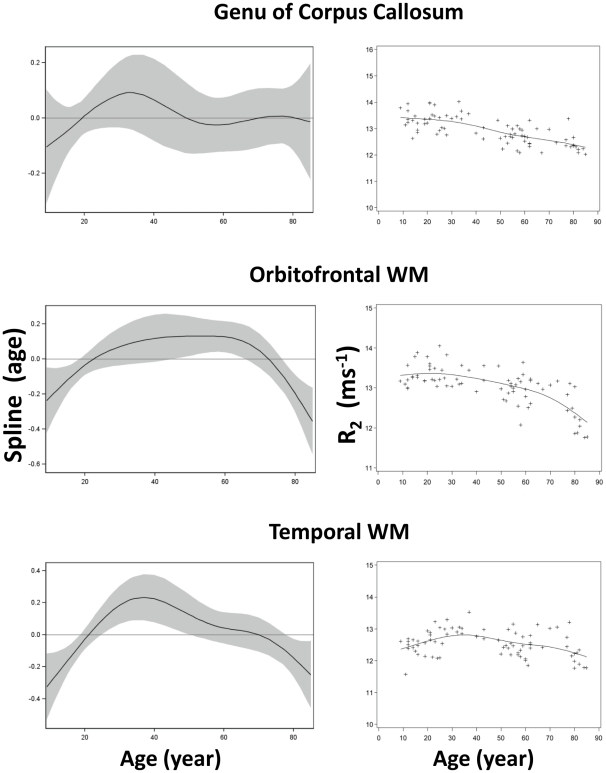
Scatter plots and fitted curves of R_2_-age correlations in corpus callosum, orbitofrontal and temporal white matter. Graphs in the left column are partial predictions plots of estimated smoothing spline functions against age with a 95% confidence band for the whole curve; graphs in the right column plot the predicted values of R_2_ against age (solid line) with the observed data overlaid (plus signs). Top, genu of corpus callosum; middle, orbitofrontal WM; bottom, anterior temporal WM.

**Figure 7:**
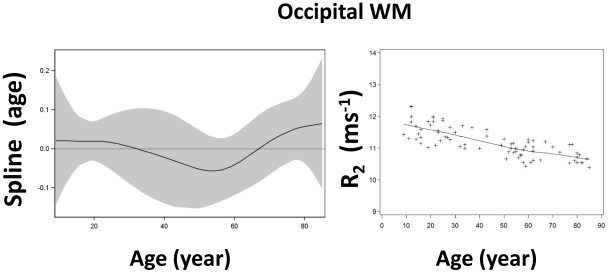
Scatter plot and fitted curve of R_2_-age correlation in the occipital white matter. Graph on the left side is a partial prediction plot of estimated smoothing spline function against age with a 95% confidence band for the whole curve; graph on the right side plots the predicted values of R_2_ against age (solid line) with the observed data overlaid (plus signs).

Among the twenty-five ROIs in this study, twenty-four were symmetrically located on the two hemispheres (bilateral) while one in the midline of the brain. Among these twenty-four bilateral ROIs, no significant difference in R_2_ between the two corresponding ROIs on each hemisphere (paired t-test, p>0.14) was observed. Thus, the R_2_ values from bilateral ROI pairs were averaged and used for subsequent analysis. The R_2_ values in Figures 3, 4, 5, 6, 7 are from 13 discrete brain structures with twelve being bilateral and one along the midline. Significant correlations between R_2_ and age were observed in all brain structures examined (see Table 2). As a general trend, the relationship between R_2_ and age was nonlinear (p<0.05) in most of the structures. Moreover, all examined gray matter structures, except the caudate nucleus, exhibited strong, nonlinear age correlations, while most white matter structures showed negative linear age correlations. These relationships are illustrated in Figures 3, 4, 5, 6, 7 and, depending on the particular structures, comprise several different patterns. For example, the R_2_ in the genu of corpus callosum and occipital white matter decreased linearly with age (p<0.001 for the linear component and p>0.17 for the nonlinear component) (see Figures 6 and 7). In these cases, the corresponding plots of the smoothing spline functions lie within the 95% confidence band containing the zero axis over the entire age range. For the remaining structures some portion of the 95% confidence band lies outside of the zero axis, indicating non-linear age correlations. The R_2_ vs. age plots for most of the gray matter structures (e.g., the hippocampus, amygdala, globus pallidus, thalamus, red nucleus and substantia nigra) showed a quadratic pattern where R_2_ increases during adolescence and young adulthood (<30 years), plateaus in middle age (30 to as early as 40 or as late as 60 years, depending on the structures), and finally, decreases in older age (Figures 3, 4, 5). In the putamen and caudate nucleus, the R_2_-age correlation appears to follow a logarithmic pattern that continues to increase after adolescence, but at a slower rate (Figures 4 and 5). In contrast, most of the white matter structures studied (e.g., the genu of corpus callosum, bilateral orbitofrontal and occipital white matter) showed a significant descending trend between R_2_ and age (Figures 6 and 7).

**Table 2:** Linear and non-linear correlation between R_2_ and age in the brain.

Structure		Linear		Non-linear	
	β (year^−1^·sec^−1^)	t-value	p-value	Chi-square	p-value
Amygdala	0.0047	4.5	<0.0001	65.56	<0.0001
Hippocampus	0.0017	2.44	0.017	101.18	<0.0001
A_Thalamus	−0.0078	−5.09	<0.0001	80.05	<0.0001
P_Thalamus	0.0024	1.00	0.32	54.13	<0.0001
Globus Pallidus	0.0054	1.77	0.081	40.09	<0.0001
Putamen	0.0353	11.19	<0.0001	27.59	<0.0001
Caudate Nucleus	0.0205	8.37	<0.0001	8.66	0.013
Red Nucleus	0.0034	1.2	0.2344	54.85	<0.0001
Substantia Nigra	0.0131	3.9	0.0002	41.09	<0.0001
G_Corpus Callosum	−0.0160	−9.01	<0.0001	3.60	0.17
Orbitofrontal_WM	−0.0138	−8.61	<0.0001	19.58	0.0004
A_Temporal_WM	−0.0042	−2.39	0.0192	19.77	<0.0001
Occipital_WM	−0.0152	−10.76	<0.0001	2.51	0.29

Note: A, anterior; P, posterior; G, genu; WM, white matter.

Table 3 shows the average T_2_ values of thirteen brain structures in normal adults. A significant heterogeneity in the T_2_ distribution in the brain was observed. The average T_2_ of these structures in twenty-six 30–59 year-old healthy normal subjects varied from 60.58±2.21 ms (globus pallidus) to 100.34±1.29 ms (hippocampus). When the sample age range was extended to 20–85 years, the average R_2_ varied from 61.31±2.42 ms (globus pallidus) to 101.60±2.25 ms (hippocampus). No significant gender difference in T_2_ for the brain structures studied was shown (for the age range 30–59 years, two-sample t-test, p>0.24; for the age range 20–85 years, two-sample t-test, p>0.07).

**Table 3:** Brain T_2_ (ms, mean/standard deviation) in adults.

Age Range	Amy	Hip	CN	GP	Pu	RN	SN	A_Tha	P_Tha	G_CC	Orbfr_WM	A_Tem_WM	Occ_WM
20–85 years (n = 65)	96.92/2.19	101.60/2.25	80.41/3.75	61.31/2.42	71.09/4.22	71.33/3.35	66.313.38	81.73/3.36	72.00/3.10	77.79/3.12	77.07/3.11	79.66/2.74	90.36/3.35
30–59 years (n = 26)	95.90/1.78	100.34/1.29	81.16/2.65	60.58/2.21	70.67/3.51	70.20/3.00	65.05/3.12	80.65/2.21	71.19/2.63	77.38/2.80	76.48/2.07	79.02/2.23	90.66/2.64

Note: A, anterior; P, posterior; Amy, amygdala; Hip, hippocampus; CN, head of caudate nucleus; GP, globus pallidus; Pu, putamen; RN, red nucleus; SN, substantia nigra; Tha, thalamus; G_CC, genu of corpus callosum; Orbfr, orbitofrontal; WM, white matter; Tem, temporal; Occ, occipital.

## Discussion

This study presents the maturational and aging effects on transverse relaxation in representative human brain structures at 3 T. The results provide needed normative data for clinical examinations and research studies utilizing transverse relaxation at this field strength. Compared to the 3 T data published previously, the transverse relaxation times acquired in this study are similar to the results reported by Wansapura et al. [15], but significantly shorter than those from other reports [7], [13], [17], [18]. Most of the T_2_ data previously published [13], [17], [18] were collected using the dual-echo method, which tends to estimate a longer than reality relaxation time. The T_2_ data in Wansapura's study were acquired with a multi-echo sequence, however, they were only acquired from a single slice, which are not sufficient to determine a quantitative baseline for general human brain studies [15]. The data from Gelman et al. [7] were obtained using a novel “Gradient-Echo Sampling of Free Induction Decay and Echo (GESFIDE)” pulse sequence designed to simultineously measure both T_2_ and T_2_^*^ [24]. Although innovative, the GESFIDE method leads to a systematically shortened T_2_ compared to those measured with the conventional, multiple spin-echo sequence on clinial systems. In the GESFIDE sequence, a long inter-echo delay (98 ms) was used to acquire a spin-echo and a series of gradient echoes during the inter-echo delay with multiple strong readout gradients. The source of the difference in T_2_ is likely from the enhanced T_2_ sensitvity to the static magnetic inhomogeneity due to water diffusion by the applied gradients during the inter-echo delay by the GESFIDE method. Bartha et al. investigated the mechanism of T_2_ relaxation in the human brain measured with multi-echo spin-echo method. Their study demonstrated that the loss of phase coherence of water magnetization due to local static magnetic field gradient could not be refocused by the 180° pulse if a significant water diffusion present during the inter-echo delay [25]. In fact, the T_2_ relaxion rate depends on the square of inter-echo delay time between acquisitions of spin-echo images for T_2_ measurement. The inter-echo delay for our measurement was 11.8 ms compared to delays as long as 98 ms in other studies. Thus, from a mechanistic point of view, apparent T_2_/R_2_ values could vary significantly depending on the imaging sequence used and acquisition parameter settings. For the purpose of general clinical applications where measurement reproducibility and general availability are important, our apparent T_2_ measurement from the brain was conducted with a commonly used multi-echo spin-echo sequence with minimum inter-echo delay.

An important implication related to the above issue is the underlying mechanism of T_2_ change with age demonstrated by this study. T_2_ relaxation depends on water molecule mobility and microscopic magnetic environment that, in turn, depends on histological and physiological factors in the tissues and can be altered by pathological changes. As discussed earlier, depending on the specific brain structures, imaging acquisition parameters, such as echo time and inter-echo delay, should also be considered for data interpretation. For example, T_2_ relaxation measured using longer inter-echo delay is more sensitive to the local static magnetic field gradients associated with tissue conditions such as iron contents, particularly, in the iron-rich regions (e.g., substantia nigra). Conversely, shorter echo-delay would lead R_2_ to be more dependent on tissue cellularity changes in the brain structures such as white matter where water molecule diffusions are more restricted. In our study, the shortest possible inter-echo delay (11.8 ms) was used.

With respect to specific brain structures and the range of age, our data exhibited varied R_2_-age relationships. From 9 to 30 years of age, significant positive correlation between tissue R_2_ and age was observed in all of the nine gray matter structures studied (i.e., amygdala, hippocampus, anterior and posterior nucleus of thalamus, globus pallidus, putamen, caudate nucleus, red nucleus, and substantia nigra). The R_2_-age correlation after age 40, however, demonstrated more diverse characteristics. In the anterior nucleus of thalamus, R_2_ increases with age and reaches its maximum at about age 40, and decreases steadily in the older age. It is interesting to note that R_2_ reaches its maximum in the hippocampus at a much later age, at about age 60, indicating its more dynamic characteristic over the life span. In the iron-rich brain structures (globus pallidus, red nucleus, and substantia nigra), the downward trend in R_2_ after age 40 is less apparent, and in some structures (putamen and caudate nucleus), there was even an upward trend. Thus, the characteristics of R_2_-age correlations identified, particularly in iron-reach structures, are likely a result of two major contributing effects: 1) decreasing cellularity that decreases R_2_, and 2) increasing iron content that increases R_2_. A postmortem study showed that non-heme iron concentration in the brain increases with age [26], which would lead to an increase in R_2_ accordingly [27]. The increase in iron concentration with age in the putamen and caudate nucleus has reached a level that its contribution to R_2_ becomes so significant even though our R_2_ mapping method is relatively less sensitive to tissue iron contents because of the short inter-echo spacing. Thus, tissue iron likely plays an important role in the changes of transverse relaxation in the iron-rich brain structures during normal aging.

The relationships between R_2_ and age showed quadratic depedence in most gray matter structures studied, which is consistent with the observation by Hasan et al. in a study of normal subjects aged 15–58 years [17]. In the white matter structures, R_2_ appears to follow a general downward trend with less apparent quadratic age-correlation than those in gray matter. The change in R_2_ in these brain structures could reflect the more dominant changes in tissue cellularity and/or myelination during maturation and aging processes. T_2_ in white matter is known to consist of multiple components significantly influenced by the structure of myelin. They are generally classified into three components: 1) a fast relaxing component (T_2_∼10–50 ms) from the water located within the myelin sheath, 2) an intermediate component (T_2_∼55–110 ms) from intracellular and extracellular water in the tissue, and 3) a long component (T_2_>1 s) from cerebrospinal fluid [28]. The fast component of R_2_ from myelin water needs to be measured using special sequences with short echo-times (<10 ms) and very long echo-trains (>32 echoes) [29], [30]. Relevant to standard clinical studies on 3 T scanners, the apparent R_2_ measured in this study was an average of all three components weighted by TE settings (TE 11.8 to 106.2 ms with a spacing of 11.8 ms).

Growth and development of the human brain are known to occur not only in childhood, but also much later during adolescent and adult years, and such developmental trajectories vary on different timelines in different brain structures. Accordingly, the data presented here demonstrate that brain tissue R_2_-age correlations are predominantly non-linear in most brain structures while specific structures may follow significantly different time courses. Our results emphasize the importance of applying a neurodevelopmental and aging perspective to the study of neural imaging during adolescence and adulthood. The detailed age dependence of R_2_ curves established here provide a foundation for clinical studies using transverse relaxation brain mapping.
